# Performance of the Toddler and Infant (TANDI) Health-Related Quality of Life Instrument in 3–4-Year-Old Children

**DOI:** 10.3390/children8100920

**Published:** 2021-10-15

**Authors:** Janine Verstraete, Andrew J. Lloyd, Jennifer Jelsma

**Affiliations:** 1 Department of Paediatrics and Child Health, Division of Pulmonology, University of Cape Town, Klipfontein Road, Rondebosch, Cape Town 7700, South Africa; 2 Acaster Lloyd Consulting Ltd., London WC1X 8NL, UK; Andrew.lloyd@acasterlloyd.com; 3 Deparment of Health and Rehabilitation Sciences, Division of Physiotherapy, University of Cape Town, Anzio Road, Observatory, Cape Town 7925, South Africa; jennifer.jelsma@uct.ac.za

**Keywords:** TANDI, Health-Related Quality of Life, proxy, toddler, preschooler, child

## Abstract

The Toddler and Infant (TANDI) dimensions of Health-Related Quality of Life assess ‘age appropriate’ behaviour and measurement could be extended to older children. A sample of 203 children 3–4 years of age was recruited, and their caregivers completed the TANDI, Pediatric Quality of Life Inventory (PedsQL) and EQ-5D-Y Proxy. Spearman and Pearson’s correlation coefficients, and Kruskal–Wallis H-test were used to explore the feasibility, known-group validity, discriminate validity and concurrent validity of the TANDI. Children with a health condition (*n* = 142) had a lower ceiling effect (*p* = 0.010) and more unique health profiles (*p* < 0.001) than the healthy group (*n* = 61). The TANDI discriminated between those with and without a health condition. In children with a health condition, the TANDI discriminated between clinician rated severity of the health condition. The TANDI had moderate to strong correlations with similar PedsQL and EQ-5D-Y items and scores. The TANDI is valid for children aged 3–4 years and is recommended for children with a health condition, whereas the PedsQL may be better for healthy children. The TANDI is recommended for studies with young children whereas the EQ-5D-Y Proxy is recommended for a sample including older children or for longitudinal studies with preschoolers. Further work on the TANDI is recommended to establish test-retest reliability and responsiveness.

## 1. Introduction

The global burden of disease reports a great vulnerability in children under five years of age [1]. The prioritisation of interventions targeting this age group has increased considerably in the last two decades [2,3,4], and appropriate outcome measures need to be identified to monitor the impact of these interventions. Major regulatory bodies, including the United States (USA) Food and Drug Administration (FDA) and The United Kingdom (UK) National Institute for Health and Care Excellence (NICE) [5], recommend that, in addition to other health or clinical outcomes in paediatric submissions, measurement of Health-Related Quality of Life (HRQoL) should be included. There has been a concomitant interest in measuring and valuing HRQoL in children under five years in recent years [6,7,8].

There are many generic measures available to measure HRQoL in children younger than five years [6,9,10]. The Pediatric Quality of Life Inventory (PedsQL) is one of the more commonly used generic health instruments which has versions available across childhood [6,9,10]. It was developed from the World Health Organisation core health dimensions to be used across the paediatric population [11,12,13]. There are versions available for infants (1–12 months), young children (2–4 years and 5–7 years), children (8–12 years) and adolescents (13–18 years). The PedsQL has been used to measure outcomes of children across a range of conditions and settings including developmental delays [14,15], toddlers with very low birth weight [16], post burn injury [17], different levels of physical activity [18] and those exposed to political violence [19]. The PedsQL scoring algorithm is a summation of the items included on the measure and reference can be made to general population or country data [13]. One of the limitations of the PedsQL and other generic measures is that currently they do not have any societal preference-based scores for economic evaluations.

The development and testing of preference-based measures are of particular value in economic evaluations and decision making. Preference-based measures elicit a societal-preference-based score, which allows for the calculation of quality-adjusted life-years (QALYs). QALYs are quantified on a scale ranging from 0 (death) to 1 (full health) with equal intervals allowing for losses and gains to be aggregated [20]. A review of NICE appraisals identified that there have been several appraisals submitted for review in this age group and it is anticipated that this will continue or increase in the future [8]. The applications reviewed most often included adult preference-based measures and rarely consider the view of the child or their family [8]. Further evidence is required on performance of HRQoL measurements, especially preference-based measures, in young children. This will ensure that regulatory bodies, scientists and clinicians can make an informed recommendation for inclusion of a HRQoL measure. To our knowledge, there are currently four preference-based measures available for children under five years: the Health-Related Quality of Life Utility Measure for Pre-School Children (HuPs) [21,22,23,24], the Infant Health-Related Quality of Life Instrument (IQI) [25,26,27], the EQ-5D-Y Proxy [28,29] and The Toddler and Infant (TANDI) Health-Related Quality of Life measure [30,31,32].

The HuPs was based on the Health Utilities Index (HUI) and is recommended for children aged 2.5–5 years. It was developed in Canada and Australia and includes 12 items including emotion, hearing, speech, ambulation, dexterity, learning and remembering, thinking and problem solving, pain, behaviour, general health, self-care and vision [21]. The earlier version of the instrument has been used to measure the outcome of children admitted to intensive care as infants [22,23,24], children with neuroblastoma [33,34,35] and in a Dutch community sample [36]. We were unable to identify a preference-based score for the measure. The IQI was developed in multi-national sample (China–Hong-Kong, UK, USA, New Zealand, Singapore) and is recommended for infants aged 1–12 months. The IQI has an associated preference-based scores. However, it is unclear if it is appropriate to extend the age range of the IQI to include toddlers or pre-school children, as it focuses on sleeping, feeding, breathing, stooling, mood, skin and interaction [25,26,27].

The EQ-5D-Y Proxy version was developed by adapting the adult version of the EQ-5D-3L to include youth-friendly wording and examples for the five dimensions included in the adult version (mobility, self-care, usual activities, pain or discomfort and emotions). The measure is recommended for self-complete in children from age 8 years and proxy completion from 4–7 years [29]. However, there is limited evidence of its performance as a proxy measure in young children [37]. The international protocol for valuation of the EQ-5D-Y has been published [38], and national value sets developed [39,40], but little is known about how the preference-based score will perform in children younger than the recommended age of 8 years.

The TANDI is a new measure developed for proxy completion for children aged 1–36 months [30,31] that is amenable to preference-based valuation. It was developed in South Africa on the basis of a review of the literature and stakeholder input, including international expert opinion. It is similar in presentation to the EQ-5D-Y Proxy, but dimensions were developed from the bottom up. The dimensions include movement, play, pain, relationships, communication and eating. The dimensions are norm-referenced by defining each as being ‘at an age-appropriate level’. The dimension of Pain includes a reference to observable pain behaviour of inconsolable crying, restless movement and grimacing. The norm referencing of the dimensions means that they may be applied across a broader age range, although initially only tested on young children. To explore the effect of extending the target age range, this study aims to test the feasibility and validity of the TANDI in children aged 3–4 years.

## 2. Materials and Methods

### 2.1. Participants

Children 3–4 years of age attending pre-schools and a tertiary paediatric hospital in Cape Town, South Africa were recruited. The tertiary paediatric hospital manages children in a 300-bed in-patient facility and in specialist paediatric out-patient clinics. The pre-schools included in this study accept typically developing children, some of whom may have minor health conditions. The pre-schools were from the same referral region as the hospital. The pre-schools were randomly selected from schools recommended by the department of education. The children’s HRQoL questionnaires were completed by their parent or caregivers.

**Figure 1:**
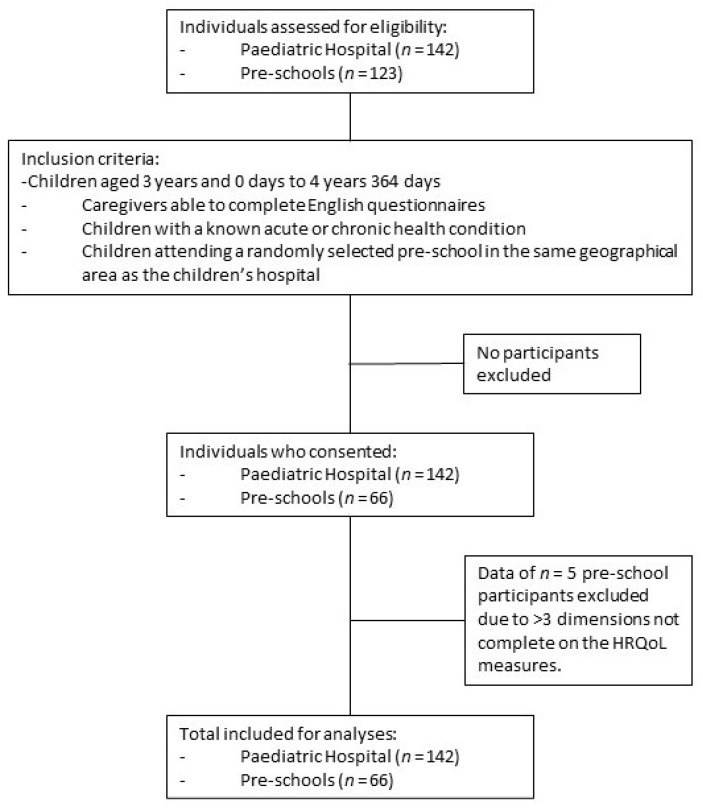
Flow diagram outlining participant inclusion process.

Participants were recruited according to their birthday and included from the day they turned three years of age until the day before their fifth birthday. Caregivers who were able to complete English questionnaires were included, as some of the measures are not available in local South African languages. Children at the paediatric hospital were eligible for inclusion if their children had a known acute or chronic health condition. All children attending the selected pre-schools were eligible for inclusion. Children who were in the intensive care unit were excluded as they were considered medically unstable or critically ill and participation in the study would be distressing (Figure 1).

The sample size was calculated to identify a difference in proportions of problems reported on the TANDI dimension scores, with a small effect size (0.4), between those with and without a health condition. A sample of 60 children was required in each group ensuring 90% power and a significance of 0.05.

### 2.2. Measures

#### 2.2.1. Toddler and Infant (TANDI) Health-Related Quality of Life Measure

The TANDI was developed for children aged 1–36 months for proxy completion [30,31]. It consists of six dimensions including Movement, Play, Pain, Relationships, Communication and Eating. The dimensions are scored across three levels of severity (no problems, some problems, or a lot of problems), and general health is scored on a Visual Analogue Scale (VAS) from 0 (worst) to 100 (best). Problems on the TANDI are described, similarly to the EQ-5D instruments [29], by a six-digit code. For example, the TANDI health state 111223 describes someone with no problems with Movement, no problems with Play, no problems with Pain, some problems with Relationships, some Communication and a lot of problems with Eating. The best health state described by the instrument is coded as 111111, describing ‘no problems’ in each of the dimensions. Thus, the TANDI has 729 (3^6^) unique health states. The TANDI does not have a preference-based score; therefore, a level sum score (LSS), similar to that used on the EQ-5D, was used to describe the responses on the descriptive system where the level labels are treated as numeric data with the best possible score (1 + 1 + 1 + 1 + 1 + 1) = 6 and the most severe score is (3 + 3 + 3 + 3 + 3 + 3) = 18 [41]. The TANDI was designed to be amenable to developing preference weights in the future.

#### 2.2.2. EQ-5D-Y

The EQ-5D-Y Proxy version 1 is a youth-friendly instrument requiring the respondent to rate the child’s health from their own (proxy) perspective [29]. The youth measure has five dimensions: Mobility, Looking after Myself, Usual Activities, Pain or Discomfort and Worried, Sad or Unhappy. Each of the dimensions is rated on a severity scale of ‘no’, ‘some’ or ‘a lot’ of problems. Proxy respondents further rate the child’s global health on a VAS between 0 and 100 (worst to best health) [28,42]. At the time of data analysis, there were two published EQ-5D-Y preference-based scores available for Slovenia [39] and Japan [40]. The Slovenian preference-based scores were used in this study.

### 2.3. Pediatric Quality of Life Inventory (PedsQL)

The PedsQL generic core scales includes proxy versions for toddlers aged 2–4 years [43]. The PedsQL includes four dimensions: physical-functioning (8 items), emotional-functioning (5 items), social-functioning (5 items) and school-functioning (5-items). Each of the 23 items are scored on a Likert scale from 0 (never a problem) to 4 (almost always a problem). All item scores are reversed and converted to a scale between 0 and 100, i.e., 0 = 100, 1 = 75, 2 = 50, 3 = 25, 4 = 0. Scores for the dimensions are computed by the summation of item scores over the number of items. The total PedsQL score, which gives an overall HRQoL score, is similarly calculated by the sum the dimension scores divided by four (number of dimensions). A higher PedsQL score suggests a better HRQoL.

### 2.4. Procedure

Ethical approval was obtained from the University of Cape Town, Human Research Ethics Committee of the Faculty of Health Sciences (HREC: 825/2017) before the study commenced. Approval was gained from all of the relevant authorities. Children were either recruited from the children’s hospital on the day of their scheduled out-patient appointment or during admission to the in-patient facility. Healthy children were recruited from pre-schools through information flyers that were sent home to the parents/caregivers.

The researcher provided the parents/caregivers with a detailed explanation of the study and the caregivers who consented were included in the study. Caregivers completed the measures with paper and pencil on the same day. The treating clinician was further asked to categorise the child’s health state on that day as mild, moderate, or severe.

A research pack was sent home with children attending the pre-schools. This included an explanation of the study, informed consent and the HRQoL measures for completion (TANDI, PedsQL and EQ-5D-Y Proxy). The caregivers were requested to return the completed research packs to school within one week if they wished to participate.

## 3. Data Analyses and Management

Children with acute or chronic health conditions attending the paediatric hospital were collapsed into a single group, labelled health condition, and compared to healthy children recruited from the pre-schools. As the group of children with acute and chronic illness was heterogeneous, expected differences could not be hypothesised. However, it was anticipated that healthy children would report a better HRQoL.

The frequency of TANDI responses to each dimension were compared across the two groups with Chi-square (*x*^2^) or Fisher exact statistics. The ceiling and floor effects were categorised as those who reported no problems (111111) or a lot of problems (333333) across all six TANDI dimensions. The proportion of reporting was compared between those with and without a health condition. The LSS score was calculated to summarise the TANDI dimension scores. The Slovenian value set, which ranges from −0.691 to 1.000, was used to calculate the index score of the EQ-5D-Y. The known-group validity was assessed for the TANDI median LSS, and the VAS score using the Mann–Whitney U-test. The discriminate validity of the TANDI was determined across those with a health condition categorised as mild, moderate, or severe by the Kruskal–Wallis H-Test. Spearman’s correlation coefficient was used to establish the concurrent validity of the PedsQL and EQ-5D-Y dimension responses. Pearson’s r was used to explore the concurrent validity between TANDI LSS and the VAS score, and EQ-5D-Y Proxy preference-based score and summary scores on the PedsQL. Correlation coefficients were interpreted according to Cohen: 0.1–0.29 low association, 0.3–0.49 moderate association and ≥0.5 high association [44].

Caregivers were asked which of the questionnaires were best able to describe the health state of their child. The proportion of their responses was compared across those with a health condition and those without with Chi-square (*x*^2^).

## 4. Results

### 4.1. Descriptive Statistics

Two-hundred and three children and caregivers, from the hospital and pre-schools, were recruited (Figure 1). All 142 caregivers at the hospital who were approached agreed and consented to participate. Across the three English medium pre-schools included, there were 123 children aged between 3 and 4 years. Research packs were distributed to all 123 children; however, only 66 were returned. Although all caregivers signed consent, data from five children in the healthy group were excluded, as three or more dimensions on the HRQoL measures were not completed. There were no exclusions due to English literacy, or for any other reasons. The data of 203 children were analysed.

The participants were categorised by health condition (attending a health facility for treatment of an acute illness (*n* = 87, 61%) or chronic illness (*n* = 55, 39%)) and typically healthy. Children in the healthy group had minor health conditions including allergies, eczema and a common cold (acute infection) that still allowed pre-school attendance (Table 1). There were no significant differences in the age, gender, or relationship of the proxy respondent to the child between those with and without a health condition (Table 1).

### 4.2. Instrument Performance and Feasibility

There were no missing values on the TANDI. The reporting of “no problems” in each of the dimensions (111111) was 34% (*n* = 70) across the total sample, as expected the healthy group showed a higher ceiling effect (*n* = 40, 28%) than those with a health condition (*n* = 30, 49%) (Table 2). Those from the healthy group reported fewer problems across all dimensions compared to those with a health condition.

No respondent reported the “pit” state (333333), and no floor effect was evident. Few reported the most severe problems. The healthy respondents did not report a “lot of” problems in any of the dimensions except for Communication, and the proportion of those reporting problems in this dimension was higher for those with a health condition.

The TANDI has 729 (3^6^) unique health profiles. Those with a health condition reported significantly more unique health profiles (*n* = 65, 46%) than those from the healthy group (*n* = 16, 26%) (*x*^2^ = 30.12, *p* < 0.001). The most common profile across groups was 111111 (Table 1) thereafter for those with a health condition 211111 (*n* = 8, 6%), 112111 (*n* = 7, 5%) and 111112 (*n* = 5, 4%) and those in the healthy group 111112 (*n* = 8, 13%), 111112 (*n* = 4, 7%) and 111222 (*n* = 4, 7%).

### 4.3. Known Group Validity

There was a significant difference between those with and without a health condition across all dimensions, except for Communication (Table 2). Healthy children reported more problems than those with a health condition for the dimensions of Relationships. The TANDI LSS and VAS scores were able to differentiate between those with and without a health condition.

### 4.4. Discriminate Validity

Children attending the health institution for an acute or chronic illness were rated by their attending health professional as being in a mild, moderate or severe health state at the same time as their caregiver completed the TANDI.

**Figure 2:**
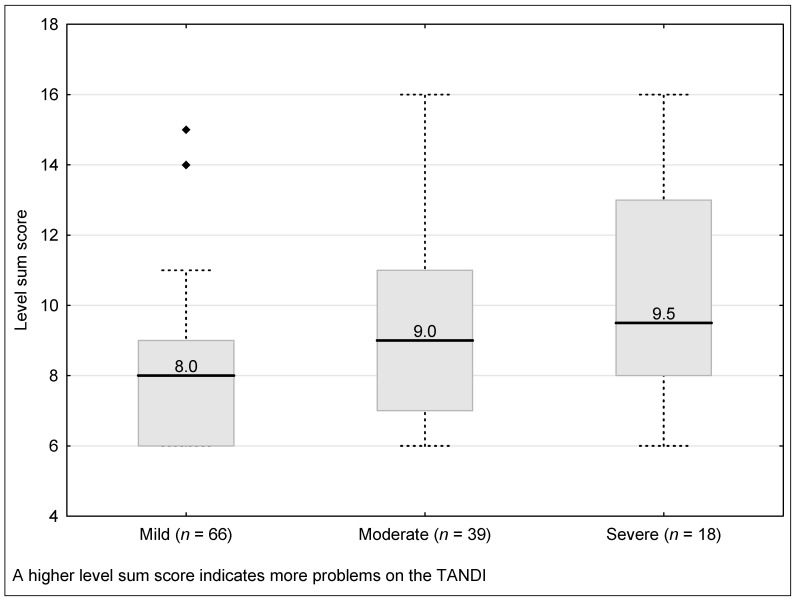
Discriminate validity of the TANDI Level sum score across the severity of health condition (Total Sample N = 203). Health condition indicates attendance at a paediatric hospital for management of an acute or chronic health condition. Boxes indicate first to third quartiles, the dividing line the median, whiskers the non-outlier range and markers the outliers.

The TANDI LSS was significantly different between those categorised as having a mild, moderate, or severe health condition (Kruskal–Wallis H = 12.61, *p* = 0.002). Those classified as mild had a significantly better LSS than those classified as moderate (Kruskal–Wallis H = −2.61, *p* = 0.009) and severe (Kruskal–Wallis H = −3.05, *p* = 0.002). However, there was no difference between those classified as moderate and severe (Kruskal–Wallis H = −0.10, *p* = 0.378) (Figure 2).

**Figure 3:**
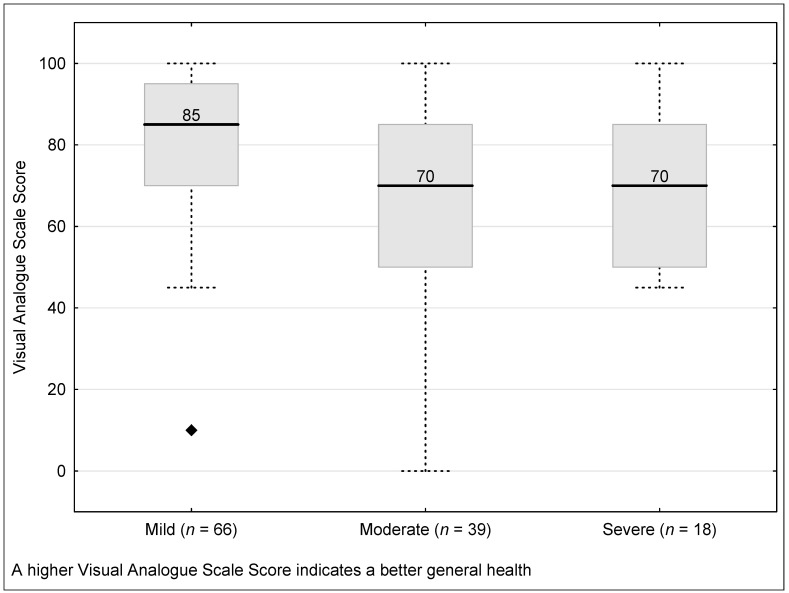
Discriminate validity of the TANDI Visual Analogues Scale score across severity of health condition. (Total Sample N = 203). Health condition indicates attendance at a paediatric hospital for management of an acute or chronic health condition. Boxes indicate first to third quartiles, the dividing line the median, whiskers the non-outlier range and markers the outliers.

The TANDI VAS score was significantly different between those with a health condition categorised as mild, moderate, or severe (Kruskal–Wallis H = 10.28, *p* = 0.006). Those classified as mild had a significantly better LSS than those classified as moderate (Kruskal–Wallis H = 1.84, *p* = 0.002). The median VAS score was higher for those classified as mild compared to severe and tended toward significance (Kruskal–Wallis H = 3.04, *p* = 0.065). There was, however, no difference between those classified as moderate and those classified as severe (Kruskal–Wallis H = −0.43, *p* = 0.667) (Figure 3).

### 4.5. Concurrent Validity of the TANDI and PedsQL

It was hypothesised that the TANDI dimension of Movement would be associated with PedsQL items of walking, running and, active play or exercise and EQ-5D-Y Proxy dimensions of mobility. The TANDI dimension of Play would be associated with PedsQL items of active play or exercise and the EQ-5D-Y Proxy dimension of usual activities. Pain on the TANDI and pain or discomfort on the EQ-5D-Y Proxy would be associated. The TANDI dimension of Relationships would be associated with the PedsQL social functioning sub-score. Communication on the TANDI was anticipated to show an association with the PedsQL sub-score of the same school activities as peers. Neither the PedsQL nor the EQ-5D-Y Proxy had any items or dimensions that were anticipated to show an association with Eating on the TANDI. A degree of agreement between all TANDI and EQ-5D-Y Proxy is expected, as both instruments have three levels of report. Thus, high levels of agreement are expected for similar dimensions.

There were no missing responses on the PedsQL items, and one missing response in the dimension of looking after myself on the EQ-5D-Y-Proxy. Children with and without a health condition reported having problems on each of the sub-scales of the PedsQL. The performance of the EQ-5D-Y-Proxy is presented elsewhere [38].

Table 3 indicates that TANDI dimensions of Movement, Play, Relationships and Communication are all moderately associated with PedsQL Total. Moderate to high correlations were seen with items that were hypothesised to be similar on the PedsQL, except for Relationships that showed a significant, but weak, association with the social-functioning sub-score. Communication showed a moderate correlation with the item of same school activities as peers. TANDI dimensions of Movement, Play and Pain were highly associated with EQ-5D-Y Proxy dimensions of Mobility, Usual Activities and Pain respectively.

The TANDI LSS score had a low to moderate association with PedsQL sub-scores and was highly associated with the PedsQL total score and the EQ-5D-Y Proxy preference-based score (Table 4). The TANDI VAS score showed a low and moderate association with the PedsQL total score and EQ-5D-Y preference-based score respectively.

### 4.6. Caregivers’ Opinions of the TANDI, PedsQL and EQ-5D-Y Proxy

Caregivers were asked which of the questionnaires they felt were best able to describe the health state of their child. Table 5 shows that children with a health condition felt that the TANDI was best able to describe their child’s health, whereas those without a health condition felt that the PedsQL better captured their child’s health condition. There was no difference in preference between those aged 3 years and 4 years (*x*^2^ = 0.27, *p* = 0.447). No reasons for their answers were recorded.

## 5. Discussion

This was the first study to extend the age range of the TANDI to children older than 3 years. The inclusion of norm referencing of the dimensions was anticipated to make the TANDI suitable for children older than the original target population of children, younger than 3 years [31]. This was found to be the case, as the instrument demonstrated feasibility and reliability in this group of children.

As no other studies reported the ceiling effect of the TANDI with reporting of problems across all dimensions (111111), comparison was made to the ceiling effect of the EQ-5D-Y in older children. The ceiling effect of healthy children and those with a health condition in this study was comparable to the EQ-5D-Y in children over eight in both the general Swedish population [45] with cystic fibrosis and functional disability [46,47], respectively. At a dimension level, the TANDI showed a similarly high proportion of ‘no problems’ in healthy preschoolers and toddlers and infants [31] compared to those with a health condition. As anticipated, the reporting of unique health profiles was higher in those with a health condition to those without, this affirms the TANDI’s ability to capture the difference in health condition and severity thereof [46]. Furthermore, there is no concentration around select health profiles, which is advantageous for a wide distribution of values in the future, and the potential for measuring a change in health state is increased [41].

The TANDI dimension of Relationships had a higher reporting of problems in the healthy group in this study whereas in younger children those with a health condition reported significantly more problems than the general population [31]. This could be attributed to reference to interaction with family members which may be more appropriate in toddlers and infants than pre-school children. Furthermore, the dimension of Communication was significantly different between health groups in toddlers and infants but not in this study, owing to the large number of healthy children reporting ‘some problems’. However, those with a health condition reported a higher number of ‘a lot of problems’. At a composite level, the TANDI showed good known-group validity and was able to discriminate between the levels of severity of the health condition. Although the severity rating by the clinician was subjective there seemed to have been a broad agreement between the caregiver and clinician. The TANDI was able to discriminate between mild and severe, which suggests that a more granular instrument might not be able to discriminate between levels that are closer together (e.g., 5 levels). It is suggested that future research explore the discriminate validity in those with disease groups that are known to differ in severity.

The TANDI showed improved concurrent validity to the PedsQL than reported for the EQ-5D-Y Proxy [37]. This could be attributed to the fact that the TANDI and PedsQL items are more similar and developmentally appropriate for children aged 3–4 years than the dimensions included in the EQ-5D-Y Proxy. In young children, the development of skills is integrated across areas of functioning resulting in more TANDI dimensions and PedsQL items showing an association than hypothesised. It was, however, evident that the PedsQL did not measure problems with Pain or Eating. Thus, for children with a health condition, where pain and/or eating is important it would be recommended to use the TANDI or EQ-5D-Y-Proxy.

It has been previously reported that the EQ-5D-Y Proxy dimensions did not perform as well in children aged 3–4 years as they did in older children [28,37]. This is not surprising as the measure was adapted from the existing adult version. The TANDI dimensions, in contrast, were identified based on input from stakeholders and literature on child development [30]. The caregiver respondents in the current study reported that the TANDI was better able to describe their child’s health if they had a health condition. Thus, the TANDI would be recommended for use in children 3–4 years with a health condition. For studies that include infants and pre-school children or for longitudinal studies for infants and toddlers, the TANDI would be a suitable and valid instrument. In a study where pre-school children with a health condition are included with older school-going children or with a longitudinal component into school-going age, the EQ-5D-Y Proxy would be recommended. However, the poor dimension performance of looking after myself, due to the developmental age of the child, as previously reported should be anticipated [37]. The PedsQL would be recommended for studies with typically developing children who report minor health impairments as its sub-scales do not have such a high ceiling effect and the caregiver respondents in this study considered it to describe the health of their child well.

### Study Limitations

The healthy group attended a pre-school in the same referral region that the paediatric hospital serves to ensure that children from similar socio-economic circumstances were recruited; the sample was, however, not matched. The results appear to reflect the age group; however, they cannot be generalised to the Western Cape or South Africa, as no data on ethnicity, language, or socioeconomic status were collected for comparison to the general population of the Western Cape. The LSS used for the TANDI is a crude summary of dimensions and indicates the performance of the instrument in the absence of utility values. The limitation of the LSS includes that different health profiles may have the same score although the severity may differ [48]; it assumes that the difference between levels of report is equal and that dimensions are equally weighted [49]. The TANDI was designed to be amenable to developing preference weights and further research is needed to determine tariffs.

## 6. Conclusions

Based on the above results, the TANDI is a valid instrument for measuring HRQoL in children aged 3–4 years with and without a health condition. The TANDI and EQ-5D-Y Proxy are recommended for children with a health condition especially if problems with pain or eating are anticipated. The PedsQL may be better for healthy children or in a cohort where a ceiling effect is anticipated. The TANDI is recommended for studies where the population includes only young children or infants and toddlers who will be included for longitudinal follow-up into pre-school. Meanwhile, the EQ-5D-Y Proxy is recommended where pre-school and school-going children are included or for longitudinal follow up of preschoolers into school-going age. Evidence on the application of EQ-5D-Y preference-based scores to children younger than 8 years is warranted. Similarly, evidence on eliciting societal preferences for very young children with the TANDI is prudent. Further work on the TANDI is recommended in additional countries and/or cultural groups and to establish performance in a larger sample of children within disease groups that are known to differ in severity and include test-retest reliability and responsiveness.

## Figures and Tables

**Table 1:** Descriptive statistics of the sample (Total Sample N = 203).

	Health Condition		Healthy		Total		*X* ^2^	*p*-Value
	(*n* = 142)		(*n* = 61)		(*n* = 203)			
	n	%	n	%	n	%		
Age								
3 years	73	51%	32	52%	105	52%	0.02	0.888
4 years	69	49%	29	48%	98	48%		
Gender								
Male	75	53%	27	44%	102	50%	0.93	0.335
Female	67	47%	34	56%	101	50%		
Relationship of caregiver to child								
Mother	115	81%	50	82%	165	81%		0.086
Father	18	13%	11	18%	29	14%		
Other *	9	6%	0	0%	9	4%		
Health condition								
None			46	75%	46	23%		
Cerebral Palsy	27	19%			27	13%		
Chronic Respiratory illness	23	16%	3	5%	26	13%		0.037
Acute Burn Wound	21	15%			21	10%		
Surgical Procedure	19	13%			19	9%		
Fracture	17	12%			17	8%		
Acute Infection ^§^	13	9%	3	5%	16	8%		0.270
Oncology	15	11%			15	7%		
Allergy			6	10%	6	3%		
Seizure requiring hospitalisation	5	4%			5	2%		
Other ꭞ	2	1%	3	5%	5	2%		

* Includes grandparents, foster parents, and adoptive parents. ꭞ Includes eczema, grommets, acute gastroenteritis and tuberculosis meningitis. Chi-square and Fisher’s exact *p*-values are presented for the difference between those with and without a health condition. ^§^ Acute infection in those with a health condition indicates hospital admission, whereas those in the healthy group reported a common cold that did not affect their pre-school attendance.

**Table 2:** TANDI dimension responses for children with a health condition and a healthy sample. (Total Sample N = 203).

		Health Condition		Healthy		Fisher’s Exact	*p*-Value
		(*n* = 142)		(*n* = 61)			
		n	%	n	%		
Movement	1	80	56%	58	95%	34.29	<0.001
	2	40	28%	3	5%		
	3	22	15%	0	0%		
Play	1	90	63%	59	97%	28.00	<0.001
	2	39	27%	2	3%		
	3	13	9%	0	0%		
Pain	1	89	63%	57	93%	21.84	<0.001
	2	47	33%	4	7%		
	3	6	4%	0	0%		
Relationships	1	114	80%	47	77%	8.54	0.011
	2	17	12%	14	23%		
	3	11	8%	0	0%		
Communication	1	103	73%	45	74%	3.56	0.168
	2	21	15%	13	21%		
	3	18	13%	3	5%		
Eating	1	100	70%	40	66%	6.12	0.038
	2	33	23%	21	34%		
	3	9	6%	0	0%		
111111		40	28%	30	49%	7.43	0.010
LSS	Median (IQR)	8 (6, 10)		7 (6, 8)		z = 2843.50	<0.001
VAS	Median (IQR)	80 (60, 95)		90 (80, 95)		z = 5634.00	0.001

N = 203. Fisher’s exact, or Mann–Whitney-U test *p*-values are presented as appropriate. 1 = no problems, 2 = some problems, 3 = a lot of problems. LSS = Level sum score, VAS = Visual Analogue Scale. TANDI VAS score is measured between 0–100, a higher score indicates better perceived general health. TANDI LSS is measured between 6 and 18, with a higher score indicating more problems.

**Table 3:** Summary table of Spearman’s correlation of TANDI dimension versus PedsQL and EQ-5D-Y Proxy. (Total Sample N = 203).

PedsQL	TANDI					
	Movement	Play	Pain	Relationships	Communication	Eating
Walking	−0.49 **	−0.30 **	−0.03	−0.18 *	−0.30 **	−0.08
Running	−0.51 **	−0.30 **	−0.07	−0.17 *	−0.28 **	−0.03
Active play or exercise	−0.44 **	−0.33 **	−0.14 *	−0.22 **	−0.31 **	−0.14
Lifting something heavy	−0.36 **	−0.23 **	−0.05	−0.14	−0.28 **	−0.02
Helping to pick up toys	−0.18 *	−0.16 *	0.02	−0.39 **	−0.39 **	−0.19 **
Sub−score Physical Functioning	−0.42 **	−0.26 **	−0.01	−0.27 **	−0.38 **	−0.12
Afraid or scared	−0.03	0.03	−0.09	−0.07	0.00	−0.08
Sad or blue	−0.22 **	−0.16 *	−0.15 *	−0.23 **	−0.10	−0.11
Angry	−0.08	−0.02	−0.06	−0.19 **	−0.16 *	−0.09
Worrying	−0.08	−0.11	−0.13	−0.08	0.03	−0.17 *
Sub−score Emotional Functioning	−0.13	−0.09	−0.14 *	−0.18 *	−0.08	−0.12
Playing with other children	−0.15 *	−0.10	−0.12	−0.23 **	−0.32 **	−0.07
Others not wanting to play with them	−0.25 **	−0.21 **	−0.13	−0.06	−0.17 *	−0.08
Teased	−0.21 **	−0.21 **	−0.22 **	−0.17 *	−0.18 **	0.00
Sub−score Social Functioning	−0.26 **	−0.22 **	−0.23 **	−0.24 **	−0.34 **	−0.08
Same school activities as peer	−0.26 **	−0.23 **	0.03	−0.30 **	−0.38 **	−0.14
Missing school as not feeling well	−0.17 *	−0.16 *	−0.10	−0.15 *	−0.26 **	−0.08
Missing school for doctor or hospital	−0.17 *	−0.12	−0.01	−0.12	−0.22 **	−0.09
Sub−score School Functioning	−0.21 **	−0.15 *	−0.02	−0.21 **	−0.35 **	−0.15 *
Total score	−0.43 **	−0.32 **	−0.13	−0.30 **	−0.39 **	−0.14 *
**EQ−5D−Y Proxy**						
Mobility	0.72 **	0.55 **	0.23 **	0.29 **	0.35 **	0.11
Looking after myself	0.50 **	0.40 **	0.09	0.37 **	0.48 **	0.17 *
Usual Activities	0.52 **	0.55 **	0.29 **	0.32 **	0.35 **	0.15 *
Pain or Discomfort	0.28 **	0.31 **	0.77 **	0.04	0.05	0.15 *
Worried, Sad or Unhappy	0.18 *	0.26 **	0.33 **	0.09	−0.02	0.18 **

N = 203. Spearman’s r: * *p* < 0.05, ** *p* < 0.001, shaded cells indicate expected associations, italics indicates significant correlations and bold indicate highly significant. PedsQL item scores range between 0 and 100, with a higher score indicating better HRQoL. TANDI and EQ-5D-Y Proxy dimensions are scored between 1 and 3 with a higher score indicating more problems.

**Table 4:** Summary table of TANDI concurrent validity. (Total Sample N = 203).

	TANDI	
	LSS	VAS
PedsQL		
Physical functioning sub−score	−0.46 **	0.17 *
Emotional functioning sub−score	−0.19 **	0.21 **
Social functioning sub−score	−0.37 **	0.12
Total score	−0.50 **	0.20 **
EQ−5D−Y Proxy		
Preference−based score	−0.71 **	0.46 **

N = 203, Pearson’s r: * = *p* < 0.05, ** = *p* < 0.001, italics indicates significant correlations and bold indicate highly significant.

**Table 5:** Questionnaire which best described the health state of the child with and without a health condition. (Total Sample N = 203). Health condition indicates attendance at a paediatric hospital for management of an acute or chronic health condition.

	Health Condition		Healthy	
	(*n* = 142)		(*n* = 61)	
	*n*	%	*n*	%
TANDI	47	33%	8	13%
PedsQL	38	27%	37	61%
EQ-5D-Y	33	23%	11	18%
All of them	25	18%	5	8%

## Data Availability

The data presented in this study are openly available in FigShare at 10.25375/uct.16810555.

## Abbreviations

FDA	Food and Drug Administration
HRQoL	Health-Related Quality of Life
HUI	Health Utilities Index
HuPs	Health Related Quality of Life Utility Measure for Pre-school Children
IQI	Infant Health-Related Quality of Life Instrument
LSS	Level Sum Score
NICE	National Institute for Health and Care Excellence
PedsQL	Paediatric Quality of Life Inventory
QALYs	Quality Adjusted Life Years
TANDI	Toddler and Infant Health-Related Quality of Life Instrument
VAS	Visual Analogue Scale
